# The long-term follow-up of the living liver donors

**DOI:** 10.1007/s13304-024-01894-4

**Published:** 2024-06-26

**Authors:** Riccardo De Carlis, Gabriele Di Lucca, Andrea Lauterio, Leonardo Centonze, Luciano De Carlis

**Affiliations:** 1 PhD Course in Clinical and Experimental Sciences, University of Padua, Via 8 Febbraio, 235122 Padua, Italy; 2 Department of General Surgery and Transplantation, Azienda Socio-Sanitaria Territoriale Grande Ospedale Metropolitano Niguarda, Piazza Ospedale Maggiore 3, 20162 Milan, Italy; 3 School of Medicine and Surgery, University of Milano-Bicocca, Milan, Italy; 4 Clinical and Experimental Medicine PhD Program, University of Modena and Reggio Emilia, Modena, Italy

**Keywords:** Biliary complications, Liver transplant, Living donation, Biliary strictures, Incisional hernia, Quality of life

## Abstract

Living donor liver transplantation (LDLT) has been proposed in many countries to reduce organ shortage. While the early postoperative outcomes have been well investigated, little is known about the long-term follow-up of the living donors. We, therefore, designed a systematic review of the literature to explore long-term complications and quality of life among living donors. We searched MEDLINE and EMBASE registries for studies published since 2013 that specifically addressed long-term follow-up following living-donor liver donation, concerning both physical and psychological aspects. Publications with a follow-up shorter than 1 year or that did not clearly state the timing of outcomes were excluded. A total of 2505 papers were initially identified. After a thorough selection, 17 articles were identified as meeting the eligibility criteria. The selected articles were mostly from North America and Eastern countries. Follow-up periods ranged from 1 to 11.5 years. The most common complications were incision site discomfort (13.2–38.8%) and psychiatric disorders (1–22%). Biliary strictures occurred in 1–14% of cases. Minimally invasive donor hepatectomy could improve quality of life, but long-term data are limited. About 30 years after the first reported LDLT, little has been published about the long-term follow-up of the living donors. Different factors may contribute to this gap, including the fact that, as healthy individuals, living donors are frequently lost during mid-term follow-up. Although the reported studies seem to confirm long-term donor safety, further research is needed to address the real-life long-term impact of this procedure.

**Graphical abstract:**

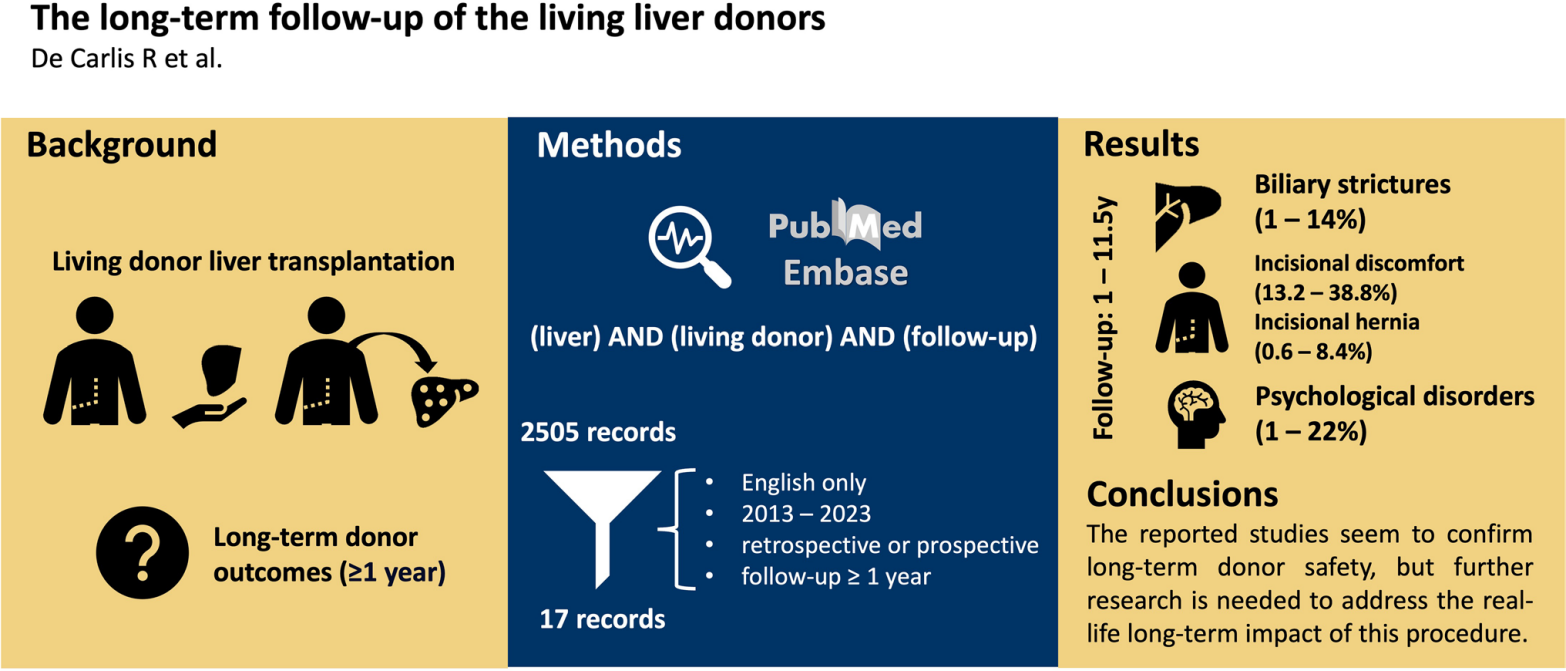

## Introduction

Liver transplantation is the definitive treatment for patients suffering from end-stage liver disease and selected cases of primary and secondary liver tumors. However, the shortage of deceased donors has resulted in many patients being unable to receive a transplant. Along with the use of livers with extended criteria from deceased donors, living donor liver transplantation (LDLT) has been increasingly proposed as a possible solution to this problem.

The first successful LDLT was performed in 1990 on a child by Strong et al., followed by the first successful transplant on an adult by Hashikura et al. in 1993 [1, 2]. Ensuring the safety of both the donor and the recipient has always been a top priority in LDLT. The use of the right liver graft without the middle hepatic vein has been classically preferred, thus balancing the safety of the donor with the need for a sufficient graft volume in the recipient [3–5]. Nevertheless, advancements in surgical technique have led to increased use of left liver grafts in the past few years, to better ensure donor safety. Minimally invasive techniques have also been adopted to reduce the impact of the donor procedure.

The safety of living donors has been extensively investigated in the context of in-hospital care and short-term follow-up. The death of a donor is the most tragic and devastating complication for the donor’s family, the recipient, and the transplant team. In Europe and the United States, the reported in-hospital rate of donor death is 0.2%, while complication rates range from 15 to 40% in the first year post-donation [6, 7]. However, despite being almost 35 years since the first liver transplant from a living donor, the long-term effects on donors remain uncertain due to several factors, such as small sample sizes, high loss to follow-up, and recall bias. Nevertheless, this information is critical to promote living donation, provide potential donors with accurate information, and assess the adherence of this practice to the Hippocratic principle of first do no harm. We have therefore carried out a systematic review of the literature to investigate long-term mortality, complications, and quality of life among donors of LDLT.

## Methods

### Study selection

This systematic review was performed following the 2020 Preferred Reporting Items for Systematic Reviews and Meta-analysis (PRISMA) guidelines. The following string was used to search from MEDLINE and EMBASE registry: ((liver) AND (living donor)) AND (follow-up). The query was performed on August 26, 2023. Publication titles, abstracts, and full-text articles were screened independently by two authors (R.D.C and G.D.L).

### Eligibility criteria

Only studies published in English in the last 10 years since 2013 that specifically addressed long-term follow-up following living-donor liver donation were included. Inclusion criteria were retrospective or prospective studies specifically related to the liver-living donors’ follow-up concerning both physical and psychological aspects. Publications with a follow-up shorter than 1 year (mean or median) or that did not clearly state the timing of outcomes were excluded. Additionally, previous reviews on the topic were removed. Abstracts, letters to the editor, case reports, and small case series (i.e., less than 20 cases) were screened for relevant information but excluded from the summary table. Duplicates and partially duplicate series were also removed.

### Data extraction

Data extracted included authors, publication year, country, type of study, number of patients, follow-up, incidence of early complications, biliary complications, incisional hernia, QoL indicators, and other long-term complications.

## Results

**Fig. 1:**
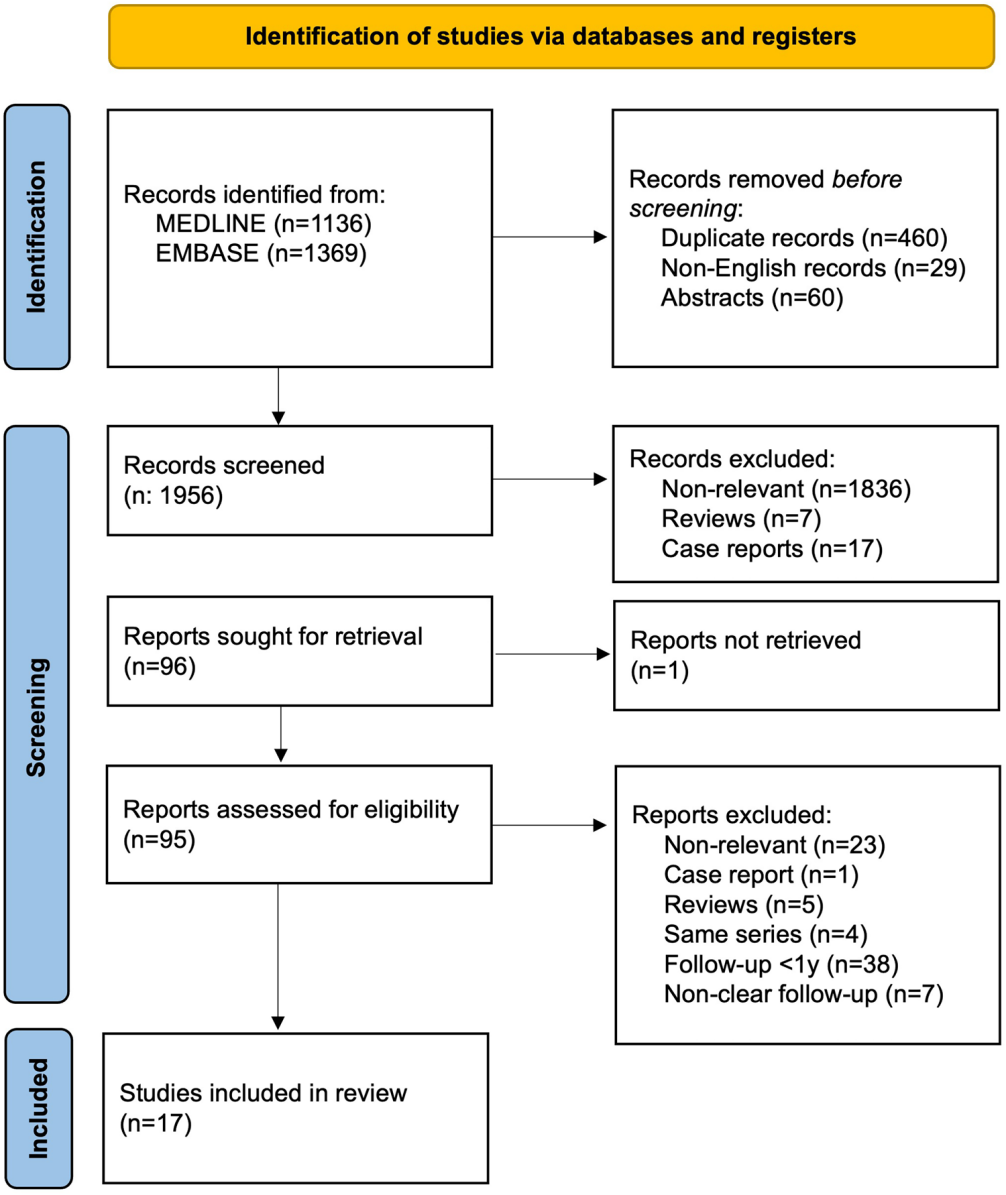
PRISMA flowchart of study selection. A total of 2505 papers were initially identified from MEDLINE (*n* = 1136) and EMBASE (*n* = 1369). After the selection process, 17 of them met the eligibility criteria and were included in this review

A total of 2505 papers were initially identified (1136 from MEDLINE and 1369 from EMBASE). Before the screening, 460 duplicates, 29 papers in foreign languages, and 60 abstracts were removed. In the first screening, 1836 papers were excluded because not relevant to the study’s intended scope. Furthermore, 7 articles were excluded as review articles, 17 as case reports, and 1 due to the unavailability of its full text. This thorough selection process resulted in 95 papers that were assessed for eligibility. In the end, 17 articles were identified as meeting the eligibility criteria for this review. A detailed PRISMA Flowchart summarises the entire process (Fig. 1).

The selected articles were mostly from North America and Eastern countries. Sampling size showed a wide range of sizes from 42 to 12,371 participants, providing a broad cross-section to be examined. Moreover, inter-study follow-up periods were quite variable, ranging from a median of 1 to 11.5 years. Each study reported different long-term outcomes. Table 1 summarizes the key characteristics of the included studies. Biliary complications (both leakage and strictures), incisional hernia, and incision site discomfort were the most frequently reported complications.

**Table 1:** Long-term outcomes of living liver donors in the selected studies

Author [ref]	Year	Country	Study type	Primary outcome	MILS	Follow-up protocol	N	Follow-up [years (SD or IQR)]	Early compl. (total)	Biliary compl.	Incisional discomfort or pain	Incisional hernia	QoL indicator	Others relevant findings
Fukuda et al. [22]	2014	Japan	Retrospective study	Clinical outcomes and QoL	–	1,3,6,12 months, then yearly	100	3.8 (2.2–6.0)	13%	1%	–	3%	SF-36	–
Darwish Murad et al. [13]	2016	US	Prospective cohort study	Long-term clinical outcomes	–	Yearly	97	5.5 (1.0–10.4)	35%	1%	18 (18.6%) incisional discomfort	–	Specifically designed questionnaire	8.1% bowel dysfunction 1% depression
Lin et al. [46]	2016	China	Retrospective cohort study	Risk of peptic ulcer disease	–	–	1333	2.96 (1.93)	–	–	–	–	–	1.2% peptic ulcer disease
Oh et al. [47]	2017	South Korea	Retrospective study	Incidence of diaphragmatic hernia	–	Yearly	336	1 (0.3 – 10.8	–	–	–	–	–	2.7% Diaphragmatic hernia
Tejura et al. [18]	2018	US	Retrospective study	Long-term imaging findings	–	–	42	2.5 (1.02–5.82)	–	–	–	–	–	60% IHDD 69% OD (all asymptomatic)
Kobayashi et al. [29]	2018	Japan	Retrospective study	Complications after donor hepatectomy	Lap.	–	51	7.3 (4.8–10)	3.6%	1.2%	–	–	–	–
Berglund et al. [15]	2018	US	Prospective cohort study	Complications after donor hepatectomy	–	Yearly	176	4.8 (2.6)	39%	2.6%	38.8% incisional discomfort	0.6%	SF-36	2.6% reintervention
Butt et al. [34]	2018	US	Prospective cohort study	Fatigue, pain, and physical symptoms	–	3,6,12,24 months	245	2 (1.8–3.4)	19%	–	13% moderate-severe abdominal or back pain	–	FACIT-Fatigue BPI-pain interference scale	–
Dew et al. [33]	2018	US	Prospective cohort study	Mental and physical health	–	Yearly	424	5.8 (1.9)	1.8%	–	26% pain interference with daily activities	–	Multiple scoring systems	22% psychiatric disorders
Raza et al. [23]	2020	US	Retrospective study	Long-term impact of liver donation	–	–	68	11.5 (5.1)	20.4%	5%	13.2% fatigue and incisional discomfort	5%	USC-DQLS and SF-36	–
Shizuku et al. [32]	2020	Japan	Retrospective study	Psychiatric disorders	–	1,3,6,12 months, then yearly	254	4 (0.5–18)	–	–	–	–	–	3.1% psychiatric disorders
Takagi et al. [16]	2020	Japan	Retrospective cohort study	Short- and long-term outcomes	–	1,3,6, 12 months, then yearly	408	7.2 (2.5–12.3)	40.4%	14%	–	2%	–	3.8% FLD 2.5% depression
Lin et al. [17]	2020	Taiwan	Retrospective cohort study	Lifetime risk of biliary tract disease	–	–	1446	2.86 (1.99)	–	3%	–	–	–	–
Abdel-Khalek et al. [24]	2022	Egypt	Prospective cohort study	Short- and long-term outcomes	–	Yearly	237	9.75 (6.58–11.7)	–	–	–	8.4%	SF-36	–
Schulze et al. [14]	2022	Saudi Arabia	Retrospective study	Outcomes of donor hepatectomy	Robotic	–	501	1 (0.4–3.2)	6.4%	5.6%	–	–	–	–
Choi et al. [41]	2022	South-Korea	Retrospective cohort study	Living donors' lifetime mortality	–	–	12371	7.9 (4.6)	–	–	–	–	–	0.7% mortality
Goto et al. [48]	2023	Japan	Retrospective cohort study	Risk of developing FLD	–	1,3,12 months, then yearly for 5yrs	212	5.6 (4.3)	–	–	–	–	–	14.15% FLD

*FLD* fatty liver disease, *IHDD* intrahepatic duct dilatation, *IQR* interquartile range, *ISN* incisional site numbness, *MILS* minimally invasive liver surgery, *OD* orphan ducts, *QoL* quality of life, *SD* standard deviation, *US* United States

### Biliary complications

Bile duct complications are a significant concern for both the donor and recipient after LDLT. However, many studies primarily focus on short-term complications, particularly bile leakage after donor hepatectomy [8, 9]. Depending on different transplant centers and types of hepatectomy performed, the incidence of donors’ bile leakage appears to range between 5 and 15% and is typically detected in the early postoperative phase [10]. Ruh et al. found that a margin of less than 5 mm from the main duct and multiple hepatic arteries increases the donors' risk of bile leak [11]. This is because division near the main duct can easily damage it, and grafts with multiple arteries require more extensive dissection, which can affect the blood supply and increase the risk of ischemic injury of the main duct.

Bile duct strictures may complicate donor hepatectomy and persist over time [11]. This condition can develop due to surgical trauma, ischemia, or inflammation during the donor procedure. It may also arise from delayed healing or the formation of scar tissue around the bile duct [12]. Having multiple ducts in the graft does not seem to increase the risk of biliary strictures in the donor, while an early bile leakage raises the risk of developing strictures in the long term [11]. The included studies reported a short-term biliary complication rate between 1 and 5.6% [13, 14]. Only in a few cases (up to 1.7% according to Berglund et al.), the complication persisted during the follow-up period. In these instances, this resulted in hospital readmissions, invasive procedures, and, in rare cases, additional surgical operations [13, 15, 16].

An intriguing paper by Lin et al. examined the lifetime risk of biliary disease after liver donation by comparing the hospital admission rate for biliary disease after liver donation with the corresponding rate in the general population. The study reported that liver-living donors have a lifetime risk of developing biliary tract disease of 49.7% (95% confidence interval: 10.8–46.1%) [17]. In a relatively small series by Tejura et al., expert radiologists reviewed long-term magnetic-resonance scans of living donors and found that up to 60% of donors had dilated intrahepatic ducts and 69% had orphan ducts. However, none of the donors reported any related symptoms [18].

### Incisional hernia and incision site discomfort

Since recent years with the advent of minimally invasive liver surgery (MILS), living donor hepatectomy was possible only through a subcostal or J-shaped laparotomy, providing a significant risk of developing an incisional hernia [19–21]. Among the included studies, incisional hernia ranged between 0.6 and 8.4% [15, 22–24]. Incisional hernia following donor hepatectomy can engender patient discomfort, and pain, necessitate hospital readmission, and potentially culminate in elective or urgent surgical intervention [25, 26]. Looking at the reintervention rate among the included studies, three patients underwent elective incisional hernia repair in the study of Raza et al., while Berglund et al. reported only one incisional hernia repair 18 months after donation [15, 23].

As well as in MILS, also in minimally invasive organ procurement, either laparoscopic or robotic, the incidence of incisional hernia seems to be decreasing [27]. It is difficult to find data on the long-term incidence of incisional hernia for living donor hepatectomy with MILS, given its relatively recent introduction [28]. Only two studies included in the analysis had a follow-up of more than one year after minimally invasive donor hepatectomy, and neither of them reported the rate of incisional hernia at the port or extraction site. [14, 29]. Nevertheless, a recent consensus conference has recommended MILS over the conventional open approach for donor hepatectomy to improve long-term incisional complications [30].

Another possible long-term complication that has been reported is incision site discomfort, which persists during the follow-up time and, in a few cases, leads to incision site surgical revision [13]. According to a survey conducted by the University of Minnesota, incisional discomfort is the most common persistent symptom experienced by liver donors, with a 34% incidence over a median follow-up of 7 years [31]. It is likely that also this complication can be reduced with MILS [30].

### Mental health outcomes, quality of life, and suicide

Given that donors accrue no direct physical benefits from the surgery they undergo, substantial attention is directed towards the assessment of physical postoperative complications. Regrettably, even over extended durations, psychological outcomes are frequently overlooked.

Shizuku et al. reported, during a 4-year follow-up, a 3.1% onset of psychiatric disorders in living donors, including major depressive disorders, panic disorders, conversion, and substance use disorders. Interestingly, the median duration from surgery to psychiatric disorders was 104.5 days and half of cases also experienced a postoperative complication, such as bile stricture. The psychiatric disorder burden leads to the need for pharmacological treatment and psychotherapy of at least 3 months, and half of the patients were still in treatment after a median follow-up of 4 years [32]. In another study from Dew et al., similar results have been observed. Depression, anxiety, and alcohol use were reported in 96 (21%) donors, at least one time during a median follow-up of 5.8 years. Moreover, the researchers investigated the possible association with postoperative recovery and found that longer post-donation hospitalization, female sex, and high BMI were predictors of psychiatric disorders [33]. Severe psychological disorders have been found to correlate with negative recipient outcomes, although not all studies support this link [33, 34].

Quality of life (QoL) can be another important outcome to assess during donors’ follow-up [35]. In the included studies, most donors reported a decrease in scores in both physical and psychological domains in the early postoperative period. However, 6 months after donation, their scores aligned with those of the general population [22, 23, 36]. In a minor group, including donors who experienced recipient death after transplant, who retrospectively told about donation regret, or who were not supported by their relatives about their choice, persistent lower scores in fatigue, chronic pain, and psychological distress were reported [13, 15, 23, 34]. The impact of MILS on QoL has also been investigated, but only two studies have used standardized questionnaires to assess QoL [30, 37, 38]. One of these studies found no significant difference in body image between laparoscopic and open hepatectomy using an upper midline incision [37]. The other study showed that QoL was significantly improved at 4 weeks after laparoscopic donor right hepatectomy compared to open surgery [38]. However, the advantage may not be sustained in the long term as there was no difference in scoring just 6 months after donation.

The occurrence of suicide among living liver donors has prompted significant concern within the medical community. In a study by Trotter et al., two suicides were reported 22 and 23 months post-donation, along with one suicide attempt, among 392 liver donors in the US [39]. Subsequently, a global survey led by Cheah et al. reported 23 deaths out of 1553 living donors, translating to an all-cause mortality rate of 0.2% during the entire follow-up. Three of these deaths (13%) were attributed to suicide, one occurring two months after donation, and the other two occurring 4 and 5 years later [40]. The etiology of these suicides cannot be directly linked to liver donation, as underlying issues or predispositions may have existed before donation or could be attributable to other causes. Nevertheless, the incidence of suicides reported by Trotter et al. was significantly higher compared to the national rate in the US [39]. More recently, Choi et al.’s study in Korea further emphasized this concern, reporting two suicides within 6 months and 3 additional cases within 1 year in a cohort of 12,371 donors [41]. Moreover, this study revealed a higher risk of death from intentional self-harm among liver donors compared to matched healthy controls [HR 1.94 (1.21–3.09)]. Yet, given that suicide is a leading cause of death among individuals under 40 in Korea, caution is warranted in interpreting these findings.

### Pregnancy after living donation

Although the pregnancy of liver transplant recipients has been reported in detail, pregnancy in living donors has not yet been thoroughly studied. In 2007, Lin et al. reported a living donor left lateral segmentectomy in a pregnant woman at 18 weeks of gestation. The recipient was her 1-year-old child. The postoperative course was uneventful, and the mother gave birth to a healthy term baby without any complications 5 months later [42]. Soon after, Soyama et al. reported 2 pregnancies within 6 months of right lobe donation without complications [43].

According to a Japanese survey investigating all LDLT cases in Japan in 2003, sexual dysfunction or menstrual irregularity has been reported in 1.7% and 2.7% of the cases, respectively. Anxiety about pregnancy or delivery among female donors has also been reported [44]. In a recent multi‐institutional survey of 6 US transplant centers including 276 women who underwent living liver donation, one-fifth of women who attempted pregnancy after liver donation reported infertility. However, the majority (74%) eventually went on to successful live births, and, aside from increased reporting of abnormal liver enzymes and cesarean deliveries, there was no significant difference in pregnancy outcomes before and after living liver donation [45].

## Conclusions

About 30 years after the first reported LDLT, little has been published about the long-term follow-up of the living donors. Different factors may contribute to this gap, including the fact that, as healthy individuals, living donors are frequently lost during mid-term follow-up. Long-term mortality rates for living donors are similar to those of the general population, though recent evidence indicates that survival outcomes may be worse than those of healthy individuals. The main sources of morbidity in the long term are incisional discomfort and psychological disorders. The incidence of these conditions varies extensively among different studies, and, as in the case of psychological disorders, it is often difficult to link them directly to the donation. Nevertheless, living donors should receive ongoing medical and psychological care after donation. MILS for donor hepatectomy could potentially improve QoL and reduce incisional discomfort. However, data on the long-term follow-up are still limited. Further studies are needed to address the real-life long-term impact of living liver donation.

## Data Availability

Data are available upon reasonable request to the corresponding author.

## Abbreviations

LDLT: Living donor liver transplantation
MILS: Minimally invasive liver surgery
QoL: Quality of life
